# Pre-Sternal Abscess associated with Deep Neck Infection

**Published:** 2014-09-01

**Authors:** Yasemin Ozsurekci, Eda Karadag Oncel, Berna Oguz, Fatma Neslihan Ayvaci, Ates Kara

**Affiliations:** 1 Department of Pediatric Infectious Diseases, Hacettepe University Faculty of Medicine, Ankara, Turkey; 2 Department of Radiology, Hacettepe University Faculty of Medicine, Ankara, Turkey; 3 Department of Pediatrics, Hacettepe University Faculty of Medicine, Ankara, Turkey

**Dear Sir,**

Deep neck space infections (DNSI) involve deep fascial space in the head and neck area and is a challenging problem because of variable clinical manifestations.[1] Accurate diagnosis and effective treatment of DNSIs is critical for successful management because of their rapidly progressive nature in children.

A previously healthy 2-year-old girl presented with a 2-day history of fever and right cervical lymphadenitis. History revealed acute otitis media two weeks ago. Physical examination of the head, neck, and oropharynx revealed hyperemic oropharynx. Her white blood cell count (WBC) and C-reactive protein (CRP) were 15400 109/L and 8.7 mg/L, respectively. The patient was hospitalized with a diagnosis of deep neck infection and empiric antibiotic treatment with sulbactam-ampicillin was started. The blood culture was negative. A pre-sternal mass of 10 cm in diameter was noticed on fifth day of hospitalization [Fig. 1]. The posteroanterior chest radiograph showed air in the soft tissue at the level of right cervical and interclavicular region [Fig. 2 (a), (b)]. Further imaging with contrast-enhanced computerized tomography (CT) of the neck and thorax demonstrated subcutaneous edema and soft tissue swelling with large abnormal air collections suggesting abscess formation from the right cervical region to the anterior chest wall [Fig. 2 (c), (d)]. Due to rapid progression even under antimicrobial treatment, antibiotic therapy was changed to meropenem, clindamycin, and vancomycin in addition to surgical incision and drainage. Twenty milliliter of pus was drained and the culture of the exudate grew Streptococcus anginosus sensitive to vancomycin. Primary immunologic work-up was found to be normal. Patient was discharged home after 15 days of hospitalization.

**Figure:**
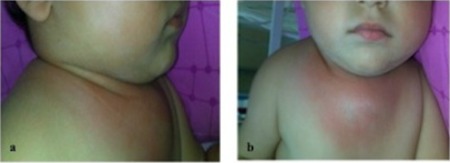
Figure 1: Deep neck infection was located mainly in the right cervical area (a), spreads and ends with a swelling (b) on anterior thoracic wall.

**Figure:**
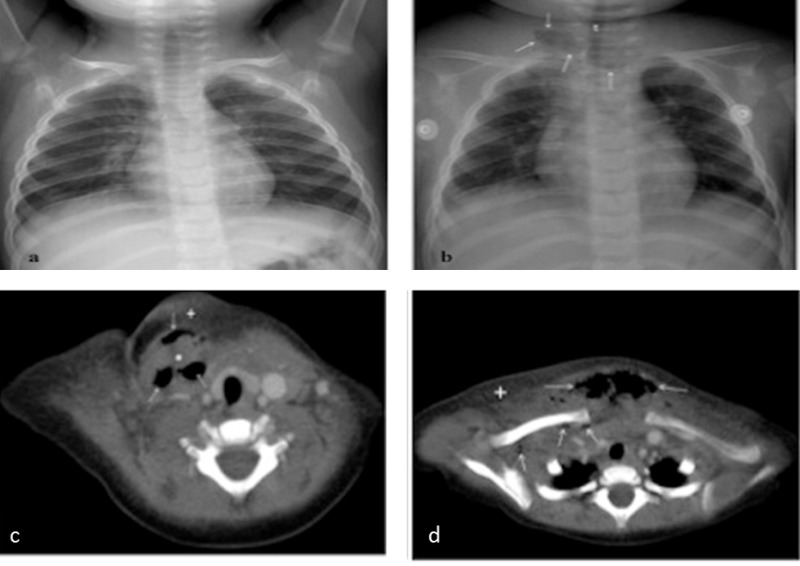
Figure 2: The chest radiography, that was normal on the referring day (a), shows air in the soft tissue (b) at the level of right cervical and inter-clavicular region on 5th day of admission (arrows). t: trachea. Contrast-enhanced axial CT shows an inflammatory swelling of the soft-tissue (*) with large abnormal air collections (arrows) suggesting abscess formation in the right cervical area (c), and spreads into the inter-clavicular region of the anterior chest wall (d). There is also subcutaneous edema (+)

Deep neck space infection often starts as cellulitis of the isolated area soft tissue adjacent to the source of infection such as oropharyngeal or cervical infectious sources. Deep neck space infections usually originate from teeth, pharynx, salivary glands, middle ear, or mastoid areas through spreading along facial planes.[2] The condition may be fatal unless promptly recognized and aggressively treated. Although we could not demonstrate the exact source of infection, oropharynx may be the original portal of entry or this condition may be a late complication of otitis media. The fascial layers of the neck and natural defense mechanisms help to prevent spread of these infections. However, if the infection is not adequately treated, a severe lymphadenitis in the lymph nodes draining the primary infection site or cellulitis in the soft tissues may progress to a purulent fluid collection called abscess.[3] The anatomy of this area is complex, and includes multiple potential spaces with connecting pathways down to the mediastinum. Infection can spread easily from one potential space to another and to connecting regions too.[4] It is probable that our patient’s pre-sternal abscess may be the result of the invasion of the infection through superficial layer of the deep cervical fascia. Additionally, while adults often have numerous localizing signs and symptoms, children with DNSI tend to have a more subtle presentation in that they are seldom able to verbalize their symptoms or cooperate with the physical examination.[5]

Adequate antimicrobial coverage, surgical drainage and appropriate management of complications remain the cornerstone for the majority of pediatric deep neck abscesses. Since there was a rapid progression to a pre-sternal abscess during intravenous antibiotic treatment, external incision and drainage with no complication was performed.

## Footnotes

**Source of Support:** Nil

**Conflict of Interest:** None declared

